# A case report of adult-onset COQ8B nephropathy presenting focal segmental glomerulosclerosis with granular swollen podocytes

**DOI:** 10.1186/s12882-020-02040-z

**Published:** 2020-08-28

**Authors:** Yujiro Maeoka, Toshiki Doi, Masaho Aizawa, Kisho Miyasako, Shuma Hirashio, Yukinari Masuda, Yoshihito Kishita, Yasushi Okazaki, Kei Murayama, Toshiyuki Imasawa, Shigeo Hara, Takao Masaki

**Affiliations:** 1 Department of Nephrology, Hiroshima University Hospital, 1-2-3 Kasumi, Minami-ku, Hiroshima, 734-8551 Japan; 2 Diagnostics and Therapeutics of Intractable Diseases, Intractable Disease Research Center, Graduate School of Medicine, Juntendo University, 2-1-1 Hongo, Bunkyo-ku, Tokyo, 113-8421 Japan; 3 Center for Medical Genetics, Department of Metabolism, Chiba Children’s Hospital, 579-1 Heta-cho, Midori-ku, Chiba, 266-0007 Japan; 4 Department of Nephrology, National Hospital Organization Chibahigashi National Hospital, 673 Nitona, Chuou-ku, Chiba, 260-8712 Japan; 5 Department of Diagnostic Pathology, Kobe City Medical Center General Hospital, 2-1-1, Minatojimaminamimachi, Chuo-ku, Kobe-city, Hyogo 650-0047 Japan

**Keywords:** Coenzyme Q8B, Coenzyme Q10, Focal segmental glomerulosclerosis, Granular swollen epithelial cells, Podocytopathy

## Abstract

**Background:**

Primary coenzyme Q10 (CoQ10) deficiency of genetic origin is one of a few treatable focal segmental glomerulosclerosis (FSGS). Renal morphologic evidence for *COQ8B* mutation and CoQ10 deficiencies of other gene mutations is assessed using electron microscopy with marked increase of abnormal-shaped mitochondria in podocytes. However, light microscopic morphologic features of deficiencies other than FSGS have not been reported.

**Case presentation:**

A 30-year-old woman was admitted to our hospital because proteinuria was found during four consecutive medical checkups. She had no medical history or family history of proteinuria and severe renal dysfunction. The swollen podocytes were stained to the same extent as mitochondria-rich proximal tubular cells under both Masson’s trichrome and hematoxylin-eosin staining, whereas no mitochondrial abnormalities were detected under the first electron microscopic views. As proteinuria and estimated glomerular filtration rate (eGFR) deteriorated after pregnancy, we reevaluated the additional electron microscopic views and detected mitochondrial abnormalities. Genetic testing revealed *COQ8B* mutation (c.532C > T, p.R178W); therefore, we diagnosed COQ8B nephropathy. CoQ10 supplementation improved proteinuria and stopped eGFR reduction.

**Conclusions:**

This is the first report of granular swollen podocytes due to mitochondrial diseases detected under light microscopy. We propose that this finding can be the clue for the diagnosis of both COQ8B nephropathy and the other CoQ10 deficiencies.

## Background

Focal segmental glomerulosclerosis (FSGS) is a leading cause of end-stage kidney disease in children and adults [1]. Steroid-resistant nephrotic syndrome (SRNS) is a glomerular disease characterized by massive proteinuria, most often associated with FSGS. Among almost 50 genes identified as causes of FSGS and/or SRNS [2–4], primary coenzyme Q10 (CoQ10) deficiency of genetic origin is one of a few treatable FSGS and/or SRNS [4–12]. CoQ10 is a mitochondrial coenzyme that is essential for the mitochondrial respiratory chain and ATP production. A set of at least 17 different genes synthesized CoQ10 in mitochondria [13–17]. Of these, mutations in ten genes (prenyl diphosphate synthase subunit 1 [*PDSS1*], *PDSS2*, *COQ2*, *COQ4*, *COQ6*, *COQ7*, *COQ8A*, *COQ8B*, *COQ9*, and AarF domain containing kinase 2) cause primary CoQ10 deficiency [17, 18]. Many signs and symptoms of CoQ10 deficient patients are common among other mitochondrial diseases; these involve multiple organ systems and often show prominent neurologic and myopathic features. However, glomerular dysfunction, such as early-onset FSGS and/or SRNS, is peculiar to CoQ10 deficiencies because of the following mutations: *PDSS2* [6, 7], *COQ2* [8], *COQ6* [9, 10], and *COQ8B* [11, 12, 19–24]. Of four genes, mutation in *COQ8B* causes selective glomerular phenotype mostly without neurological and myopathic deficits [11, 12, 19–24]. The age at onset is usually between 5 and 20 years [11, 12, 19–25].

Renal morphologic evidence for *COQ8B* mutation and CoQ10 deficiencies of the other gene mutations is a marked increase of abnormal-shaped mitochondria in podocytes as assessed using electron microscopy [8, 10, 20, 21]. In the other mitochondria diseases, the accumulation of abnormal mitochondria is more likely seen in tubular cells than podocytes under electron microscope because mitochondriopathies typically cause tubulopathy [26]. Consistent with this, granular swollen epithelial cells (GSECs) in tubular cells are easily detected under light microscope [27]; these cells are a distinct morphologic feature that suggests mitochondrial diseases. However, swollen podocytes accumulating abnormal mitochondria remain undetected by light microscope. Additionally, light microscopic morphologic features other than FSGS under CoQ10 deficiencies are also lacking. Sampling error for electron microscopic material may greatly affect the finding of sporadic abnormal podocytes; therefore, identifying abnormal podocytes by light microscopy may be helpful for suspecting COQ8B nephropathy and other CoQ10 deficiencies. Here, we report the case of a patient with homozygous *COQ8B* mutation (c.532C > T, p.R178W) who presented an adult-onset FSGS without family history. Light microscopy clearly showed granular swollen podocytes, which corresponded with podocytes filled with numerous dysmorphic mitochondria by electronic microscopy.

## Case presentation

A 30-year-old Japanese woman with no chief complaints was admitted to our hospital because proteinuria was found during four consecutive medical checkups. Urinary protein level was assessed at her medical checkup every year. She had no medical history and family history of proteinuria or severe renal dysfunction. On admission, the physical examination did not show notable abnormalities; her blood pressure was normal (110/64 mmHg) and no edema was observed. The initial laboratory evaluation (Table 1) showed a normal complete blood count; serum creatinine, 0.64 mg/dL; estimated glomerular filtration rate (eGFR), 82 mL/min per 1.73 m^2^; total protein 7.3 g/dL; albumin 4.5 g/dL. Urinalysis revealed 2+ proteinuria; urine protein creatinine ratio (uPCR) was 1.85 g/gCr. Serologic workup results were negative. Ultrasonography of the kidneys showed that they were of normal size.

**Table 1:** Patient’s laboratory characteristics on admission

Parameter	Value	(normal range)
(Urine)		
pH	7.0	
Urine protein/creatinine ratio (g/gCr)	1.85	(< 0.15)
Red blood cell (/HPF)	0	(< 5)
Oval fat body	Positive	Negative
(Blood)		
White blood cell (/μL)	5290	(3040–8540)
Red blood cell (10^4^ /μL)	445	(378–499)
Hemoglobin (g/dL)	12.6	(10.8–14.9)
Platelet (10^4^ /μL)	20.8	(15.0–36.0)
AST(U/L)	17	(13–33)
ALT(U/L)	18	(8–42)
Total protein (g/dL)	7.3	(6.7–8.3)
Serum albumin (g/dL)	4.5	(4.0–5.0)
Blood urea nitrogen (mg/dL)	8.2	(8–20)
Creatinine (mg/dL)	0.69	(040–0.70)
eGFR (mL/min/1.73 m^2^)	82	(> 90)
Na (mmol/L)	139	(138–146)
K (mmol/L)	3.7	(3.6–4.9)
Cl (mmol/L)	103	(99–109)
Calcium (mg/dL)	9.4	(8.6–10.4)
Phosphate (mg/dL)	3.0	(2.5–4.7)
Uric acid (mg/dL)	4.5	2.3–7.0)
Plasma glucose (mg/dL)	91	(70–109)
Hemoglobin A1c (NGSP) (%)	5.1	(4.6–6.2)
C-reactive protein (mg/dL)	0.02	(< 0.20)
IgG (mg/dL)	1090	(870–1700)
IgA (mg/dL)	216	(110–410)
IgM (mg/dL)	206	(46–260)
CH50 (IU/mL)	35	(30–46)
C3 (mg/dL)	99	(86–160)
C4 (mg/dL)	18	(17–45)
Anti nuclear antigen	Negative	Negative
HBs-Ag	Negative	Negative
HCV-Ab	Negative	Negative

*HPF* high-power field, *AST* aspartate transaminase, *ALT* alanine transaminase, *eGFR* estimated glomerular filtration rate, *IgG* immunoglobulin G, *IgA* immunoglobulin A, *IgM* immunoglobulin M

**Fig. 1:**
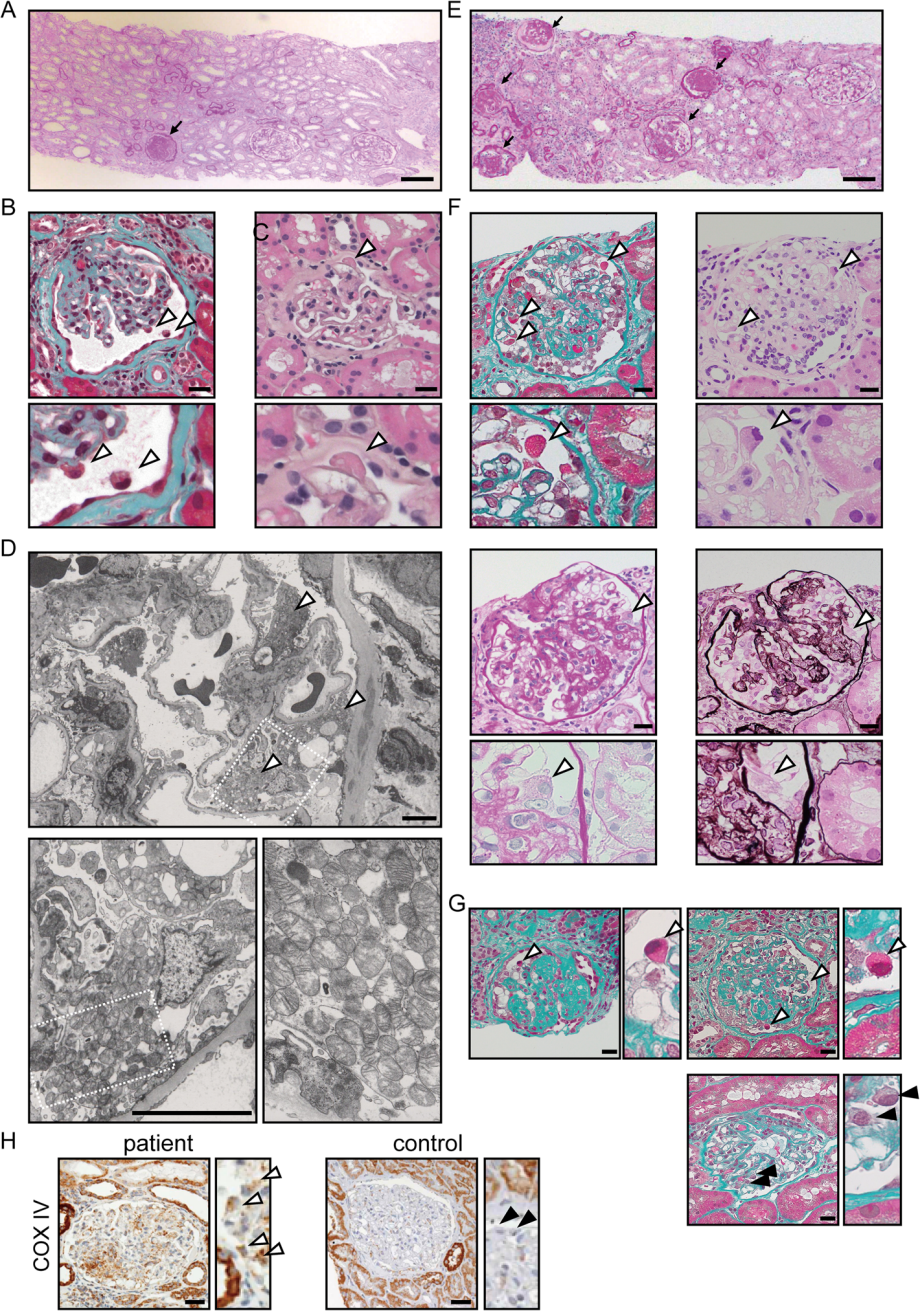
Light and electron microscopic findings at first (**a**-**d**) and second biopsy (**e**-**g**). **a, e** Periodic acid–Schiff (PAS) staining at a low magnification (×100) in the first and second biopsy, respectively. Global or segmental sclerotic glomeruli (*indicated by arrow*) were increased at the second biopsy (**e**). Scale bars, 100 μm. **b, c** Segmental sclerotic glomerular images of Masson’s trichrome (**b**) and hematoxylin-eosin (HE) staining (**c**) at a high magnification (× 400). Granular swollen podocytes were indicated by arrowheads. Scale bars: 20 μm. **d** Electron microscopic views of podocytes filled with abnormal mitochondria were observed (*indicated by arrowheads*). Segmental foot process effacement of the epithelial cells was observed (*lower left panel*). Numerous dysmorphic mitochondria lacking cristae or with abnormally enlarged ones were observed in podocyte cell bodies (*lower right panel*). Scale bars: 5 μm. **f** Light microscopic views of the granular swollen podocytes (*indicated by arrowheads*) in the same focal segmental glomerulus under Masson’s trichrome (*upper left panels*), HE (*upper right panels*), PAS (*lower left panels*), and Periodic acid–methenamine-silver staining (*lower right panels*) (× 400). Scale bars: 20 μm. **g** Granular swollen podocytes (*indicated by white arrowheads*) were observed in sclerotic glomeruli (*upper panels*), but not normal-appearing glomeruli (*lower panels*) under Masson’s trichrome staining (× 400). Normal podocytes are indicated by black arrowheads. Scale bars: 20 μm. **h** Immunochemistry showing increased segmental staining of COX IV in glomeruli from this patient (*left panel*), but not in those from the control patient with no mitochondrial disease (*right panel*). COX IV-positive podocytes and normal podocytes are indicated by white and black arrowheads, respectively. Scale bars: 20 μm

**Fig. 2:**
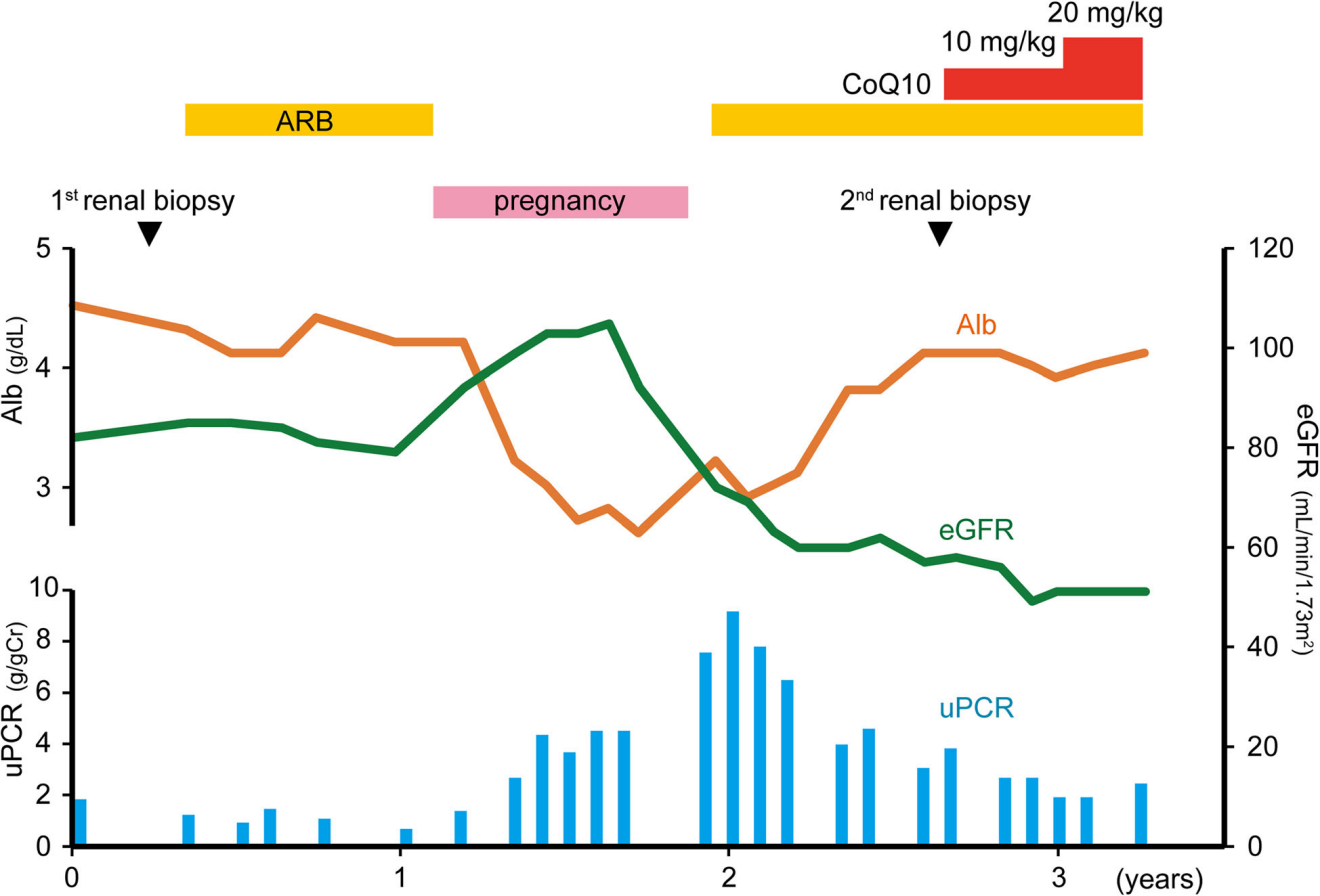
Patient’s clinical course. Changes in the estimated glomerular filtration rate (GFR, green lines), serum albumin (Alb, orange lines), urine protein/creatinine ratio (uPCR, blue bars). CoQ10: coenzyme Q10, ARB: angiotensin receptor blocker

In the first renal biopsy, 31 glomeruli, including 13 global (Fig. 1a) and 2 segmental scleroses (Fig. 1b and c), were obtained. In addition to podocyte detachment, the swollen podocytes were red-stained to the same extent as mitochondria-rich proximal tubular cells in the segmental sclerotic glomeruli under both Masson’s trichrome and hematoxylin-eosin (HE) staining (Fig. 1b and c), but the remaining glomeruli were almost normal and no significant deposition was observed in immunofluorescence. Foot processes were segmentally effaced under electron microscope, but no mitochondrial abnormalities were detected. As she did not want to examine the cause of FSGS, which had not been otherwise specified by genetic testing at this point, angiotensin receptor blocker (ARB) was started as a symptomatic treatment. This decreased her urinary protein excretion during the almost-1-year follow-up (Fig. 2). However, after ARB was discontinued for pregnancy, her uPCR increased and nephrotic syndrome developed with proteinuria, hypoalbuminemia, and decreased eGFR developed (Fig. 2). To detect mitochondrial abnormalities, we reevaluated the additional electron microscopic views, which revealed that some podocyte cell bodies were filled with numerous dysmorphic mitochondria lacking cristae or with abnormally enlarged ones (Fig. 1d). Accordingly, podocytes were also characterized by extensive foot process effacement and marked hypertrophy (Fig. 1d). Because mitochondrial abnormalities in podocytes were suspected, we analyzed genomic DNA from her peripheral blood mononuclear cells by targeted resequencing using next-generation sequencing, which revealed the existence of homozygous missense mutations c.532C > T (p.R178W) in exon 6 of *COQ8B* gene, which led to a definitive diagnosis of mitochondrial disease.

**Fig. 3:**
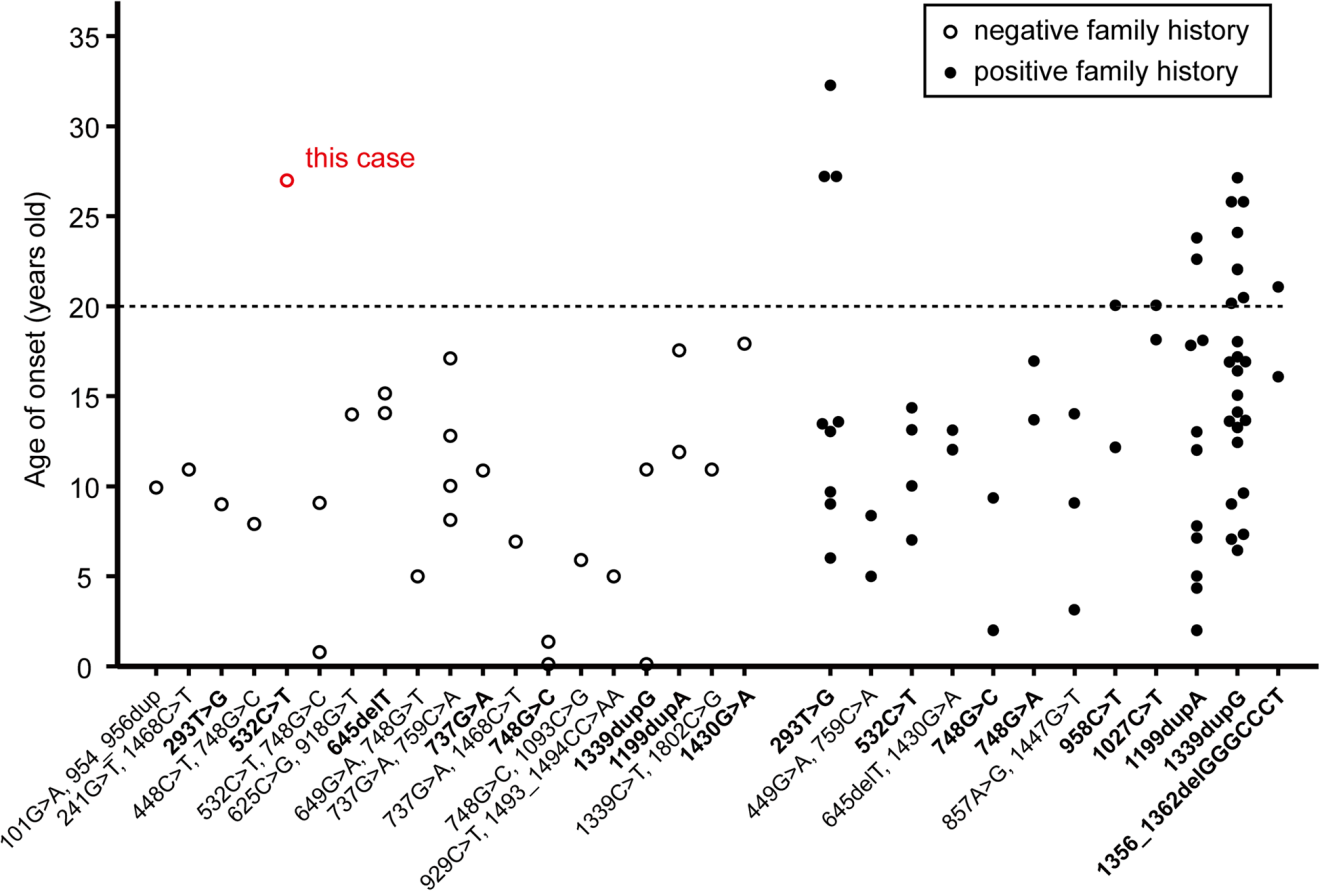
Age at onset of COQ8B nephropathy with negative or positive family history. The data were obtained from the previous reports [11, 12, 19–25] and negative or positive family histories were separately plotted as open or closed circles, respectively. This case is indicated by red open circles. Heterozygous or homozygous mutations are indicated as normal or bold letters, respectively

Before the CoQ10 supplementation, we performed a follow-up renal biopsy. In the second renal biopsy, 31 glomeruli were obtained, including 22 global (Fig. 1e) and 3 segmental scleroses, which shows the exacerbation of the percentage of glomerulosclerosis and tubulointerstitial fibrosis. As shown in Fig. 1f, Masson’s trichrome staining clearly showed granular swollen podocytes, consistent with the findings of the first biopsy. These podocytes were distinguished from protein droplets in cases with proteinuria because the absorptive protein droplets are positive for Periodic acid–methenamine-silver (PAM) staining [23]. Ten of these podocytes were observed among six glomeruli with scleroses and/or adhesion, but not among normal-appearing glomeruli (Fig. 1g). In both the first and second biopsies, no dysmorphic mitochondria were detected in other glomerular cells, including parietal, endothelial, and mesangial cells, or in myocytes of small arteries, interstitial fibroblasts, and tubular epithelial cells. After this second renal biopsy, she received treatment with oral CoQ10 at a dose of 10–20 mg/kg/day. This supplementation decreased uPCR and stopped eGFR reduction (Fig. 2).

## Discussion and conclusions

We reported the case of adult-onset COQ8B nephropathy without family history of proteinuria or sever renal dysfunction presenting with FSGS. Additionally, we showed that granular swollen podocytes were identified under light microscope and that Masson trichrome and HE staining clearly showed them. In these podocytes, protein droplets were negative for PAM stain, which suggests that they include mitochondria to the same extent as mitochondria-rich proximal tubular cells. Although the kinds of cell with mitochondrial accumulation differ, Kobayashi et al. have found GSECs among distal tubules and collecting ducts in all patients with mutated mtDNA A3243G and T3271C, and have also demonstrated that Masson trichrome stain is the best way to detect these cells because it stains mitochondria red [27]. Therefore, our light microscopic findings of podocytes due to COQ8B nephropathy appear to be similar to those of GSECs in tubules due to mitochondrial diseases [27], which suggests that this is the first report to detect GSECs in podocytes due to mitochondrial diseases under light microscopy. Moreover, increased podocyte expression of a mitochondrial-specific protein COX IV in this patient supports the observation that light microscopy can detect accumulation of mitochondria in podocytes (Fig. 1h). It might be difficult to clearly detect red-stained granules in all abnormal podocytes because their cells are smaller than tubular cells. However, under CoQ10 deficiencies, we considered GSECs in podocytes to be more important than those in tubules because CoQ10 deficiencies are more likely to cause glomerular dysfunction than tubular injury [11, 12, 19–21], as opposed to the other mitochondrial diseases [26]. Additionally, as COQ8B nephropathy may progress much more quickly than other genetic FSGS, such as *NPHS2* and *WT1* nephropathy [12], it is important to early suspect COQ8B nephropathy and properly diagnose. In our case, proteinuria rapidly increased during pregnancy and reached a nephrotic level although the deterioration might be affected by ARB discontinuation and pregnancy itself. Therefore, GSECs in podocytes appear to be a useful tool to suspect CoQ8B nephropathy and prevent their exacerbation by early diagnosis.

We observed more GSECs in podocytes in the second biopsy than the first. Both urinary protein and serum creatinine levels in the second biopsy were higher than the first, which was consistent with exacerbation of percent glomerulosclerosis and tubulointerstitial fibrosis. However, GSECs in tubular cells are uncorrelated with these parameters [27]. Interestingly, we observed GSECs in podocytes among only glomeruli with scleroses and/or adhesion, but not among normal-appearing glomeruli. This specific localization speculates that GSECs in podocytes may cause FSGS through podocytopathy. Although *COQ8B* knockdown in cultured podocytes does not affect apoptosis, loss of COQ8B reduces podocyte migration [11]. Both this impaired podocyte migration in vitro and proteinuria in clinical conditions are reversed by CoQ10 addition [11, 12, 21–24]. Consistent with these reports, treatment with CoQ10 decreased urinary protein level of our patient. Therefore, we speculate that GSECs in podocytes lead to glomerulosclerosis through podocytopathy and may be associated with both clinical and histological parameter of kidney function.

This patient had no neurological or myopathic features, which are common in mitochondrial diseases. Consistent with these observations, COQ8B mutations cause selective glomerular phenotypes, mostly without neurological and myopathic deficits [11, 12, 19–24]. Although the mechanism of this selectivity remains unclear, a potential explanation may be the difference in distribution patterns between COQ8B and COQ8A with high sequence similarity [11, 12]. COQ8B is highly expressed in podocytes, whereas COQ8A is expressed in most body tissues but not podocytes [11]. Neurologic abnormalities were reported in only 14–24% of patients with COQ8B nephropathy [12, 19]. Although we cannot completely exclude the possibility of extrarenal involvement, the patient was clinically considered to have presented a selective glomerular phenotype.

To date, 89 patients have been reported to have been diagnosed with COQ8B nephropathy and genetic analysis revealed 30 *COQ8B* mutations [11, 12, 19–25]. In this case, we found *COQ8B* mutation (c.532C > T, p.R178W) using genetic analysis. Although this mutation is a known pathogenic missense mutation [11, 12], we considered this case to be unique because the family history was negative for late-onset COQ8B nephropathy (Fig. 3). In cases with positive family history, 22.2% of patients developed COQ8B nephropathy aged 20 or older, but there were no reports of adult-onset COQ8B nephropathy in cases with negative family history (Fig. 3) [11, 12, 19–25]. One possible explanation for this difference in onset is that underdiagnosis is more likely in cases with negative family history because the locations of mutations are not strongly correlated with age at onset. In fact, frameshift mutations of c.1447G > T completely abolish COQ8B function in vitro [13], but similar frameshift homozygous mutations, such as 1199dupA, 1339dupG, and 1356_1362delGGGCCCT, do not always cause early onset (Fig. 3). Although mitochondrial diseases are diagnosed using genetic analysis or electron microscopy when it is suspected, genetic testing can be performed at only specific facilities. Thus, just as in our case, it is difficult to diagnose COQ8B nephropathy when podocytes accumulating abnormal mitochondria are not included in electron microscopic views. Therefore, it is important to carefully suspect CoQ10 deficiencies in cases in which patients with proteinuria, even if they are aged over 20 years old and lack a family history of proteinuria and severe renal dysfunction.

We cannot refer to the sensitivity or specificity of these podocyte findings because it is extremely difficult to obtain sufficient numbers of patients for rare diseases. Because Masson’s trichrome staining was not demonstrated in previous report of COQ8B nephropathy [11, 12, 19–24], further studies may be necessary to carefully evaluate and determine the scientific relevance of this staining.

In conclusion, we reported the case of patient with *COQ8B* mutation (c.532C > T, p.R178W) who presented with granular swollen podocytes in segmental and global sclerotic glomeruli under light microscope. When GSECs are found in podocytes, suspecting mitochondrial disease may lead to a faster and more accurate diagnosis. Therefore, we propose that this finding can be the clue for the diagnosis of mitochondrial nephropathy. As few patients develop COQ8B nephropathy aged 20 or older, it is important to carefully suspect CoQ10 deficiencies. Further studies using more specimens are needed to confirm our findings.

## Data Availability

The datasets during this case report are available from the corresponding author on reasonable request.

## Abbreviations

COQ: coenzyme Q
FSGS: focal segmental glomerulosclerosis
SRNS: steroid-resistant nephrotic syndrome
PDSS: prenyl diphosphate synthase subunit
GSECs: granular swollen epithelial cells
eGFR: estimated glomerular filtration rate
uPCR: urine protein/creatinine ratio
HE: hematoxylin-eosin
ARB: angiotensin receptor blocker
PAM: periodic acid–methenamine-silver
